# Rolling Nanoelectrode Lithography

**DOI:** 10.3390/mi11070656

**Published:** 2020-06-30

**Authors:** Rashed Md. Murad Hasan, Xichun Luo, Jining Sun

**Affiliations:** 1 Centre for Precision Manufacturing, DMEM, University of Strathclyde, Glasgow G1 1XJ, UK; rashed.hasan@strath.ac.uk; 2 Key Laboratory for Micro/Nano Technology and System of Liaoning Province, Dalian University of Technology, Dalian 116024, China

**Keywords:** nanolithography, uniformity, pattern direction, rolling speed, bias voltage

## Abstract

Non-uniformity and low throughput issues severely limit the application of nanoelectrode lithography for large area nanopatterning. This paper proposes, for the first time, a new rolling nanoelectrode lithography approach to overcome these challenges. A test-bed was developed to realize uniform pressure distribution over the whole contact area between the roller and the silicon specimen, so that the local oxidation process occurred uniformly over a large area of the specimen. In this work, a brass roller wrapped with a fabricated polycarbonate strip was used as a stamp to generate nanopatterns on a silicon surface. The experimental results show that a uniform pattern transfer for a large area can be achieved with this new rolling nanoelectrode lithography approach. The rolling speed and the applied bias voltage were identified as the primary control parameters for oxide growth. Furthermore, the pattern direction showed no significant influence on the oxide process. We therefore demonstrated that nanoelectrode lithography can be scaled up for large-area nanofabrication by incorporating a roller stamp.

## 1. Introduction

Nanofabrication over large areas paves the way for the commercial applications of nanotechnology [1]. Large-area nanofabrications are now being used to manufacture many devices and create innovative products for nanoelectronics, optoelectronics, nanophotonics, and other areas. Although conventional photolithography is currently the dominant method of patterning nanoscale features for the microelectronics industries, it has approached its ultimate limits [2,3]. In the last decade, extensive efforts have been devoted to alternative lithography techniques by various research laboratories. Some emerging and existing methods, such as Extreme Ultraviolet Lithography (EUVL) [4], Electron Beam Lithography (EBL) [5], Nanoimprint Lithography (NIL) [6], Directed Self Assembly (DSA) [7] and Scanning Probe Lithography (SPL) [8], have demonstrated excellent potential as promising candidates for future industrial nanofabrication. Each technique has its own strengths and limitations. Although electron beam lithography methods have relatively higher resolution, they are limited by low throughput [9]. Similarly, scanning probe lithography techniques need to increase throughput, possibly using a matrix of tips for parallel writing [10,11]. Again, extreme ultraviolet lithography, nanoimprint lithography and directed self-assembly techniques are getting closer to industrial requirements [1]. However, all these technologies are in their development phases and still need further work to overcome some challenges. EUVL has already been installed in various fabs worldwide [12]. The EUVL infrastructure still requires significant improvement in source reliability, line edge roughness (LER), and defectivity [1]. In addition, overlay, defectivity, tool design and placement accuracy remain the main concerns for NIL and DSA technologies [1,13,14,15].

**Figure 1:**
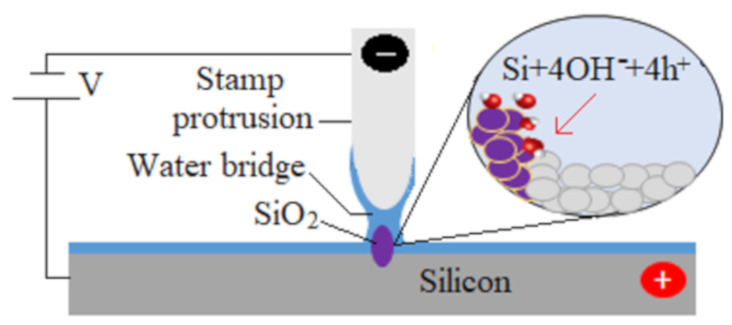
Schematic diagram of the nanoelectrode nanolithography (NEL) process on a silicon surface.

On the other hand, nanoelectrode nanolithography (NEL) has been developed in the laboratory and has been demonstrated as an efficient lithographic tool. This method is based on the spatial confinement of anodic oxidation between a conductive stamp and the specimen surface. When the conductive stamp touches the specimen, a voltage is applied between them. A water bridge is formed between the protrusive parts of the stamp and the specimen. Then the electrochemical reactions occur, which form an oxide film in the touched areas of the specimen. Figure 1 shows the schematic diagram of a NEL process. This method is robust, reliable, and flexible, and has already been effectively used to fabricate nanostructures on different specimens: silicon [16,17,18], GaAs [19,20] and pentacene layers [21]. However, the NEL process with a flat stamp is not suitable for large area nanopatterning as non-uniformity becomes an issue when they use large stamps. Firstly, it is difficult to achieve a uniform contact between the stamp and the specimen on a large-scale due to disuse of the resist materials [16]. Again, another critical challenge in the NEL process is to maintain uniform pressure over the whole contact area during oxidation. These challenges severely limit the application of NEL for large-area nanopatterning.

This paper aims to establish a new cost-effective nanoelectrode lithography technique and its test-bed for large-area nanofabrication. We propose a rolling nanoelectrode lithography (R-NEL) process, which could significantly improve the pattern transfer uniformity. This method can be advantageous as it enables continuous patterning and easy de-molding in large-area nanofabrication. The roller-based nanopatterning concepts are not new, they have been successfully utilized in thermal/UV nanoimprint lithography techniques for large-area nanofabrication [22,23,24,25,26]. These roller-based manufacturing methods have been recognized to be a high-throughput and low-cost technology. These methods can even fabricate continuous nanopatterns, which make them attractive for various industrial applications [27,28]. However, the application of a roller in NEL is rare due to the challenges in fabricating the conductive roller stamps. In this work, we will implement the proposed R-NEL system and perform the oxidation process on a Si (100) specimen. We will also investigate the influence of experimental parameters such as pattern direction, applied voltage, and rolling speed on the R-NEL process.

## 2. Experimental Methods

### 2.1. Description of the R-NEL Process

**Figure 2:**
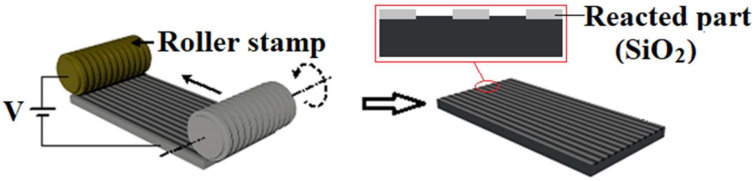
Schematic of the proposed rolling NEL system.

In the R-NEL system, the roller stamp rolls over the specimen while voltage is applied between the stamp and the specimen, which eventually produces continuous oxide patterns (Figure 2). The introduction of the roller is required to maintain uniform pressure over the whole area. Consequently, the local oxidation process can occur uniformly over a large area of the specimens.

**Figure 3:**
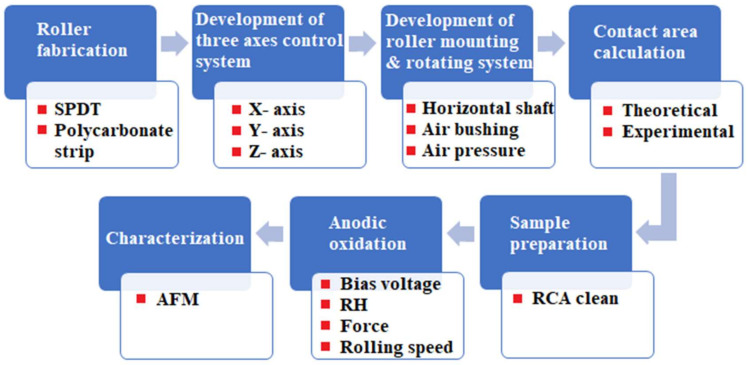
Process steps for the rolling NEL system.

A sequence of process steps was followed to develop a prototype of the R-NEL system (shown in Figure 3). The first step was to fabricate a roller with nano protrusions that can be obtained by several methods. One method is to create patterns directly on the roller surface by diamond turning or electron beam lithography processes. Another way is to wrap a belt or flexible flat stamp around a bare roller. This method is generally the easiest as the belt, or the flexible flat stamp, can easily be fabricated with various patterning techniques. However, wrapping a belt on a roller may leave a seam, which produces an inevitable joint error. In this research, we choose the latter method as it is typically more practical and cost-effective for research purposes. It is also worth noting that a seamless roller stamp should not yield any substantial technical differences, so long as the experimental parameters are the same.

**Figure 4:**
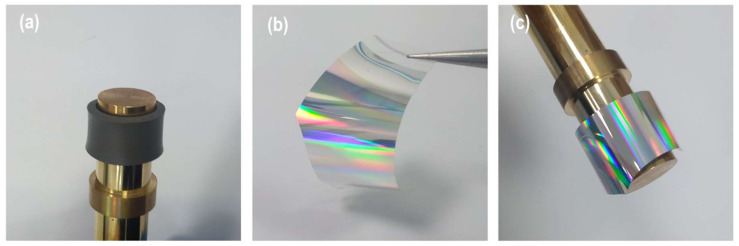
(**a**) roller with a rubber band. (**b**) peeled polycarbonate strip. (**c**) completed roller stamp.

**Figure 5:**
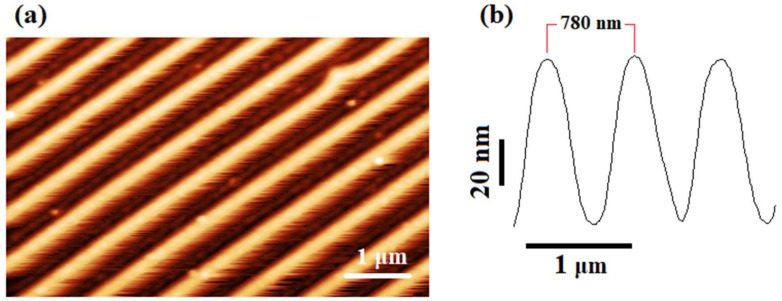
(**a**) Atomic force microscope (AFM) topographic image of the roller stamp. (**b**) Line profile of the AFM image.

In order to prepare the stamp, a cylindrical roller base was machined from brass materials on an ultra-precision diamond turning machine. The length and diameter of the roller base were selected as 100 mm and 13 mm, respectively. Then the roller base was first wrapped with a 2 mm thick and 10 mm wide layer of synthetic rubber (polyisobutylene and 2 mol% isoprene), which increases the elasticity of the stamp as well as the contact area (Figure 4a). After that, a polycarbonate strip (100 μm thick) was peeled from a DVD-R and cleaned with an ethanol solution (Figure 4b). The strip adhered to the rubber surface (Figure 4c). The polycarbonate strip was composed of spiral guide grooves with a periodicity of 780 nm, a 350 nm linewidth at half height, and a 100 nm depth. The strip was covered with a reflective metal layer of aluminum, which makes the stamp electrically conductive. The overall diameter and width of the nanostructured area of the roller became 17.2 mm and 10 mm, respectively. The atomic force microscope (AFM) image of the nanostructures showed no degradation after being peeled from the DVD and adhered to the roller (Figure 5).

**Figure 6:**
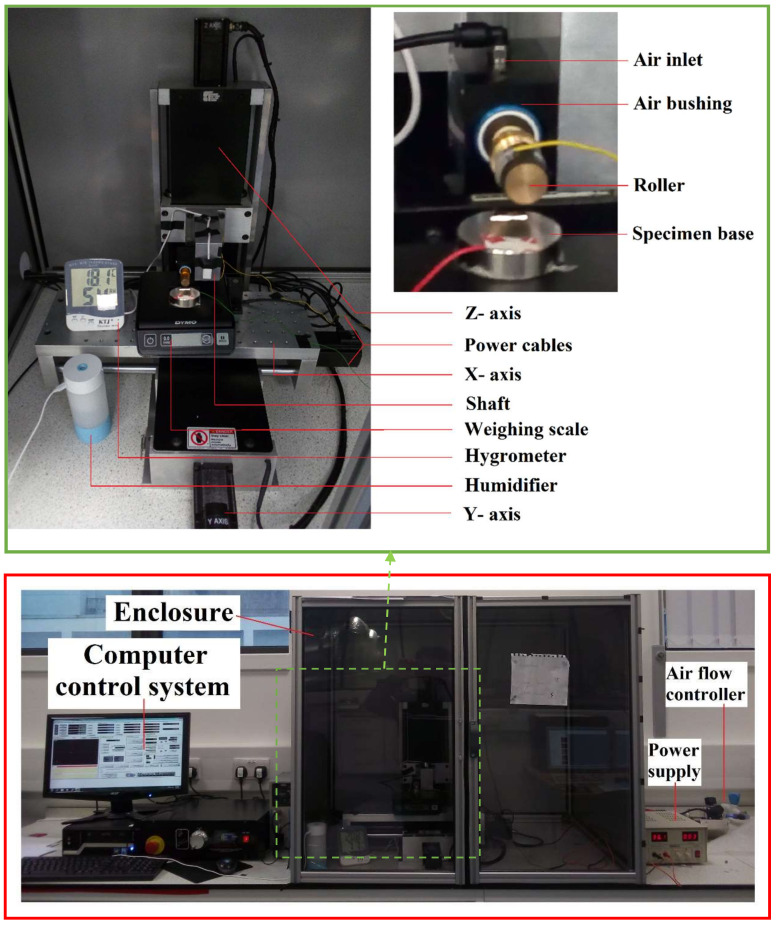
Overview of the implemented rolling nanoelectrode nanolithography (R-NEL) system.

In the next step, an air bushing (13.02 mm inner diameter) with the roller stamp was mounted on the *Z*-axis of a three-axis translational motion control system. The air bushing distributes compressed air through a tubular media containing millions of sub-micron holes, which holds the roller and enables it to rotate frictionlessly. The specimen was placed on a metal base that can move along the X and Y axes. The motions of these three axes were controlled by a computer, which allowed the correct positioning of the specimen relative to the roller stamp. The roller descended to make gentle contact with the specimen. When the specimen moved along the *X*-axis, the roller also rotated due to adhesive force at the contact between them. Thus, the roller rolled over the specimen and fabricated the whole specimen surface. A controller was used to maintain a constant airflow at the required pressure. A humidifier was used to maintain the relative humidity inside the enclosure. A hygrometer and a weighing scale were also used to measure the relative humidity and the force applied on the roller, respectively. Figure 6 shows an overview of the implemented R-NEL system.

### 2.2. Experimental Details

All the experiments were carried out at room temperature with a relative humidity of 80%. The pressure of the compressed air into the air bushing was maintained at 60 psi. An atomic force microscope (DI Dimension 3100) was used to inspect the nanostructures on the specimen surfaces. To run an experimental operation, the roller was moved towards the specimen first by using the jogging of the controller interface (Mach3) until the force reached the desired value. Then a simple program defined by a G Code was run to move the specimen in the x-direction at a specific speed.

### 2.3. Specimen Preparation

In these experiments, specimens of p-doped silicon (100) with resistivity ρ = 10–15 Ω-cm were used. These specimens were cleaned with ultrasound in an NH_4_OH/H_2_O_2_/H_2_O (1:1:5) solution for 10 min. The cleaning process was repeated thrice to achieve optimal cleaning with a low density of particles on the surface. After cleaning, the specimens were blown dry with a N_2_ gas jet.

### 2.4. Contact Area Calculation

**Figure 7:**
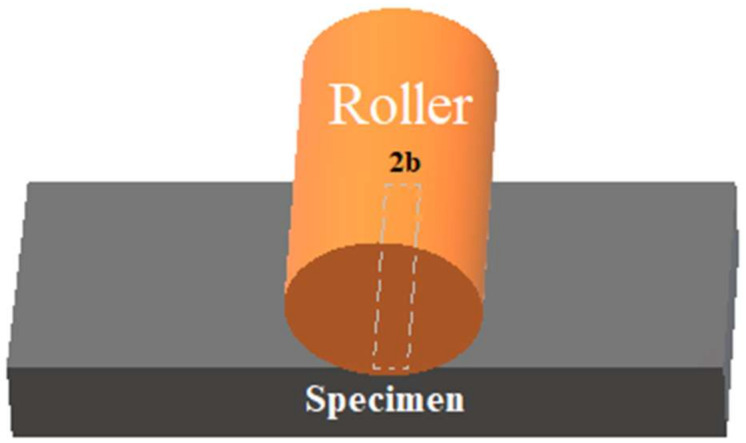
Contact area between the roller stamp and the specimen.

Obtaining a precise contact area between the stamp and the specimen is crucial to determine the rolling speed needed for the oxidation process. The actual contact area during oxidation is only a line or a finite rectangular area along the roller stamp in contact with the specimen, as shown in Figure 7. The size of the contact area, which can be determined by the Hertz theory [29], depends upon some parameters such as the applied force, roller dimensions, and the properties of the roller and the specimen.

By using the Hertz theory, the half-width (*b*) of the rectangular contact area can be written as [30]:

(1) $$b=\sqrt{\frac{4FR\left[\frac{1-v_{1}^{2}}{E_{1}}+\frac{1-v_{2}^{2}}{E_{2}}\right]}{\pi L}}$$

where *E*_1_ and *E*_2_ are the elastic moduli for the roller stamp, and the specimen and *ν*_1_ and *ν*_2_ are the Poisson’s ratios, respectively. *R* is the radius of the roller stamp, and *L* and *F* refer to the length of the contact area and the applied force, respectively.

The rolling speed can be calculated by using the following formula:

(2) $$Rolling\;speed=\frac{2b}{t}$$

where *t* is the time required for the oxidation process.

The maximum limit for the rolling speed can be obtained by considering the minimum required time for the oxidation process. To evaluate the contact area, oxidation was performed with this roller stamp for one minute without any rotation of it. By careful visualization of the oxide area with the AFM, the value appeared to be ~1.1 mm, which is very close to the calculative value of 1.15 mm (calculations are available as Supporting Information). Hence, a rolling speed of 0.50 mm/min ensures an exposure time of ~2 min.

## 3. Results and Discussions

### 3.1. Patterning by R-NEL

**Figure 8:**
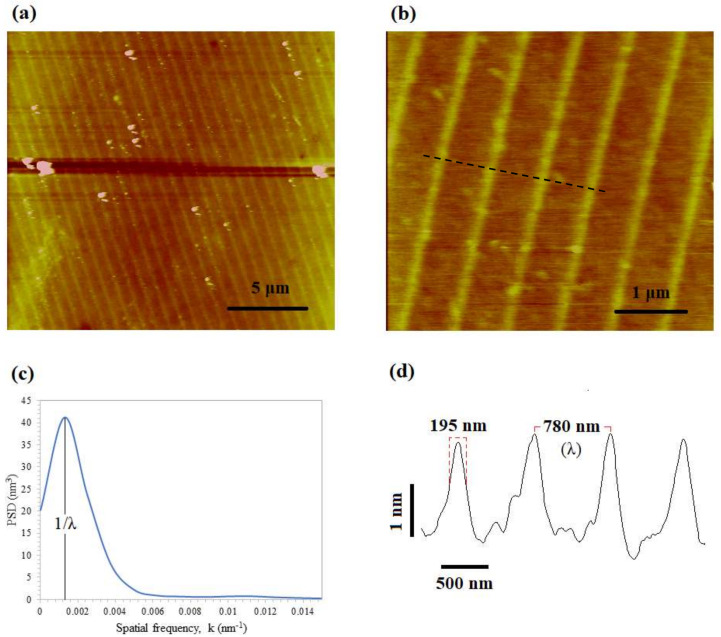
(**a**,**b**) Atomic force microscope (AFM) topography image of the oxide lines made by R-NEL. (**c**) Power spectral density (PSD) of the AFM image shown in (**b**). (**d**) Profile of the lines marked in the image (**b**).

The first experiment was carried out using the roller stamp, where the patterns were orthogonal to the moving specimen direction. Figure 8 shows the result of the oxidation after applying a 1 N force and a voltage of 36 V at a rolling speed of 0.50 mm/min. To characterize the surface morphology, we calculated the power spectral density (PSD) (shown in Figure 8c) and the roughness of the surface by using the characterization methodology proposed in [31]. The characteristic parameters are shown in Table 1. A uniform pattern transfer was achieved with an oxide mean height of 2.1 nm, a full width at half maximum (FWHM) of 195 nm and a periodicity of 780 nm on an area of about 8 mm × 10 mm. The root mean square (RMS) roughness for the oxide lines and the spaces was found to be 217.44 pm and 179.34 pm respectively, while the skewness and the excess kurtosis were estimated as 0.8775 and 0.2828. These results indicate a significant improvement in pattern uniformity compared to the other results obtained with the conventional NEL process [32,33].

It is also worth mentioning that the seam of the roller used in the experiments restricts the length of the specimen area to be patterned. However, this should not make any qualitative technical differences, as mentioned before. Therefore, it is shown that the new R-NEL system can achieve uniform pattern replication, maintaining the same periodicity.

### 3.2. Influence of the Pattern Directions

**Figure 9:**
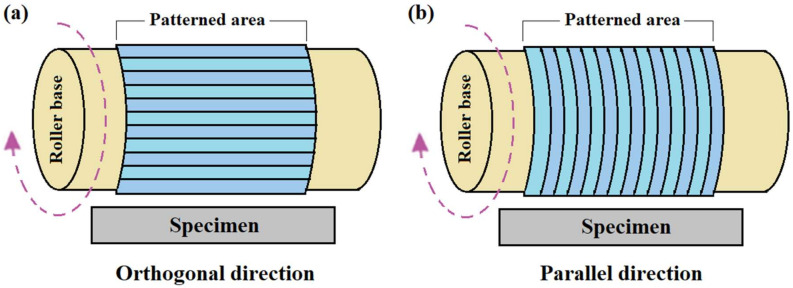
Schematic diagram of pattern directions. (**a**) Orthogonal direction; (**b**) Parallel direction.

**Figure 10:**
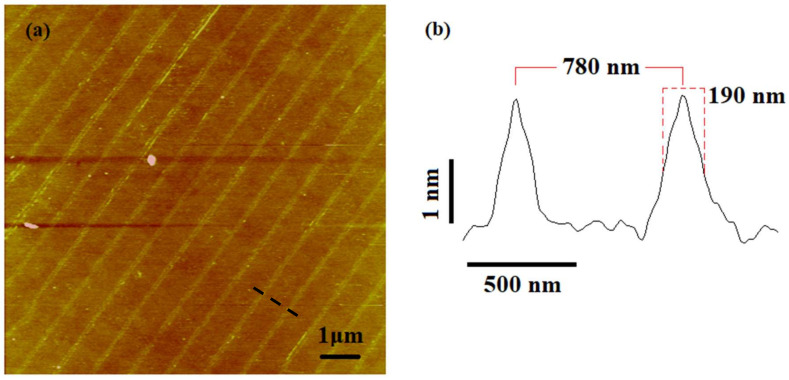
Parallel pattern direction. (**a**) AFM topography image of the oxide lines. (**b**) Profile of the lines marked in the image (**a**).

The oxidation processes performed in the previous section used roller stamps where the patterns were orthogonal to the moving specimen direction (Figure 9a). It would be interesting to see how the orientation of patterns affects the oxidation process. To do this, an oxidation experiment was carried out with the same experimental parameters where the patterns were parallel to the moving specimen (Figure 9b). An oxide pattern of 2 nm height and 190 nm FWHM were achieved while maintaining the same periodicity (780 nm) (shown in Figure 10). These oxide patterns were very similar to the pattern obtained with the roller stamp, where the patterns were orthogonal to the moving specimen direction. Therefore, it can be concluded that there is no significant influence of pattern directions on the oxidation process in the R-NEL system.

### 3.3. Effect of the Rolling Speed and the Bias Voltage

**Figure 11:**
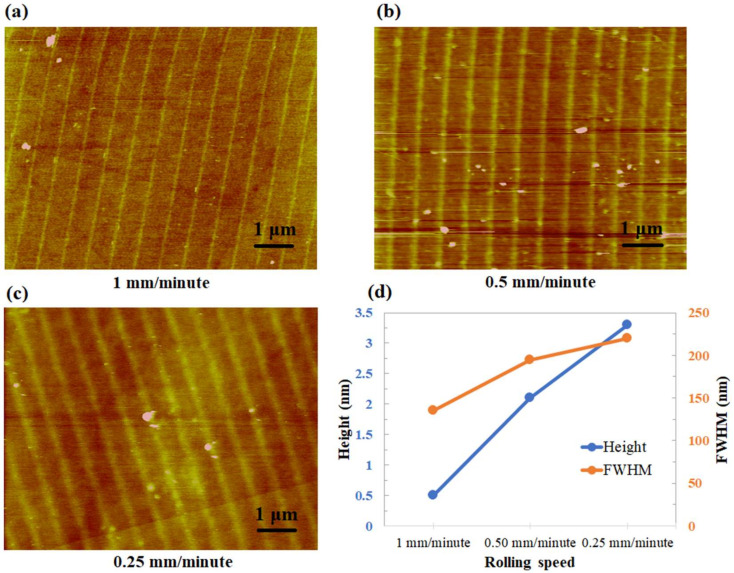
(**a**–**c**) AFM topographic images. (**d**) Relationship between the rolling speed and oxide height (left side of the graph) and full width at half maximum (FWHM) (right side of the graph).

The rolling speed and the applied voltage are two major parameters that can be altered to optimize the R-NEL process. Hence, we studied the effect of bias voltage and rolling speed on oxide growth. Applying the constant voltage (36 V), Figure 11a–c show the topographic images of the specimens for three different rolling speeds (0.25, 0.50, and 1 mm/min). The height and the full width at half maximum (FWHM) of the oxides are represented concerning the rolling speeds where each point of the graph represents an average of a few values obtained from the same specimen (shown in Figure 11d). It is shown that the oxide patterns have a linear increase in height with a decrease in the rolling speed. It can be explained by the fact that, as the rolling speed increases, the exposure time for the contact area decreases. Previous studies show that the height of the oxides increases with the oxidation time [33]. However, D. Stievenard et al. showed that the oxide height varies as log(1/speed) with the tip writing speed in the AFM oxidation lithography process [34]. In the R-NEL process, it shows linear rather than logarithmic dependency on rolling speed.

Again, it can be seen from Figure 11d that the width (FWHM) increases with decreasing rolling speed. In other words, low rolling speeds made broader oxide patterns. The growth behavior of oxide width with the rolling speed can be explained considering two models proposed for AFM oxidation. Firstly, Kuramochi et al. suggested ionic diffusion as the main reason for the lateral oxide growth during the long exposure time [35]. Ionic diffusion takes place through the adsorbed water layer when the grown oxide starts preventing the current flow under the stamp protrusion (tip). In other research, Bloeß et al. showed that the size of the water bridge limits the lateral oxide growth [36]. Although the size of the water bridge is influenced by the electric field and the relative humidity, the size of the contact area remains the main factor. Therefore, it can be concluded that the oxide width increases with the exposure time due to ionic diffusion until a certain value determined by the size of the water bridge.

**Figure 12:**
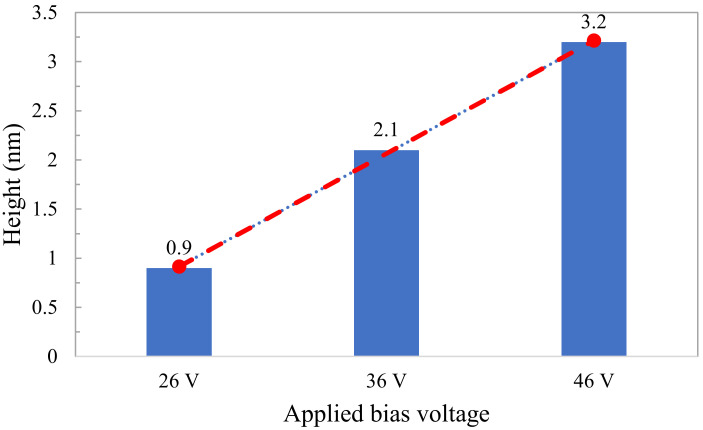
Height of oxide lines at different applied bias voltages.

In order to investigate the effect of applied voltage, the oxidation was performed with a bias voltage ranging from 26 to 46 V at a rolling speed of 0.50 mm/min. Figure 12 shows that the oxide height increases linearly with the applied voltage. This outcome is also in good agreement with the results obtained in [37]. Additionally, the aspect ratio (height/width) of the oxide lines is one of the essential factors both in mask fabrication and lithography processes for the semiconductor industry. It was found that the aspect ratio also increases as the rolling speed decreases (from 0.0037 for 1 mm/min to 0.015 for 0.25 mm/min). Therefore, precise control of the aspect ratio of the oxide lines can be achieved by careful optimization of the experimental parameters, such as rolling speed and the applied voltage. Furthermore, the throughput can easily be increased by using a roller stamp with a higher radius.

## 4. Concluding Remarks

A rolling nanoelectrode lithography approach was proposed for the first time in this paper to fabricate nanostructures on silicon specimens. A prototype system was designed and implemented where a polycarbonate strip wrapped with a brass roller base was used as the stamp. This new system requires a much smaller force since it proceeds in a small area perpendicular to the specimen moving direction. A uniform pattern transfer for the large area was achieved with this new R-NEL system. The rolling speed and the applied bias voltage were identified as the primary control parameters for oxide growth. Experimental studies show the linear dependence of the oxide height as a function of the applied voltage, whereas the oxide height is inversely proportional to the rolling speed. The effect of the pattern direction was also identified, and shows no significant differences. These results show that the new R-NEL system allows the control of the parameters involved in the oxidation process, and this could enable the use of the nanoelectrode lithography process in large area fabrication and electronic device mass production.

## Figures and Tables

**Table 1:** Metrological characteristics of the silicon surface after the NEL process.

Parameters	Value
Peak Wavenumber (1/λ) (nm^−1^)	0.001282
Periodicity (λ) (nm)	780
Average Oxide Height (nm)	2.1 ± 0.045
Average FWHM (nm)	195 ± 35
RMS Roughness in Oxide Lines (pm)	217.44 ± 33.34
RMS Roughness in Spaces (pm)	179.34 ± 7.16
Skewness	0.8775 ± 0.2854
Excess Kurtosis	0.2828 ± 1.03
